# Investigation of Colonic Regeneration via Precise Damage Application Using Femtosecond Laser-Based Nanosurgery

**DOI:** 10.3390/cells11071143

**Published:** 2022-03-28

**Authors:** Sören Donath, Leon Angerstein, Lara Gentemann, Dominik Müller, Anna E. Seidler, Christian Jesinghaus, André Bleich, Alexander Heisterkamp, Manuela Buettner, Stefan Kalies

**Affiliations:** 1 Institute of Quantum Optics, Leibniz University Hannover, 30167 Hannover, Germany; leon.angerstein@stud.uni-hannover.de (L.A.); lara.gentemann@stud.mh-hannover.de (L.G.); d.mueller@iqo.uni-hannover.de (D.M.); anna.e.seidler@stud.mh-hannover.de (A.E.S.); jesinghaus.christian@stud.uni-hannover.de (C.J.); heisterkamp@iqo.uni-hannover.de (A.H.); kalies@iqo.uni-hannover.de (S.K.); 2 Lower Saxony Center for Biomedical Engineering, Implant Research and Development (NIFE), 30625 Hannover, Germany; bleich.andre@mh-hannover.de (A.B.); buettner.manuela@mh-hannover.de (M.B.); 3 REBIRTH Research Center for Translational Regenerative Medicine, 30625 Hannover, Germany; 4 Institute for Laboratory Animal Science, Hannover Medical School, 30625 Hannover, Germany

**Keywords:** colonoid, femtosecond laser, nanosurgery, lentiviral transduction, organoid

## Abstract

Organoids represent the cellular composition of natural tissue. So called colonoids, organoids derived from colon tissue, are a good model for understanding regeneration. However, next to the cellular composition, the surrounding matrix, the cell–cell interactions, and environmental factors have to be considered. This requires new approaches for the manipulation of a colonoid. Of key interest is the precise application of localized damage and the following cellular reaction. We have established multiphoton imaging in combination with femtosecond laser-based cellular nanosurgery in colonoids to ablate single cells in the colonoids’ crypts, the proliferative zones, and the differentiated zones. We observed that half of the colonoids recovered within six hours after manipulation. An invagination of the damaged cell and closing of the structure was observed. In about a third of the cases of targeted crypt damage, it caused a stop in crypt proliferation. In the majority of colonoids ablated in the crypt, the damage led to an increase in *Wnt* signalling, indicated via a fluorescent lentiviral biosensor. qRT-PCR analysis showed increased expression of various proliferation and *Wnt*-associated genes in response to damage. Our new model of probing colonoid regeneration paves the way to better understand organoid dynamics on a single cell level.

## 1. Introduction

Understanding regeneration in an intercellular context requires models that go far beyond conventional cell culture. Intestinal tissue represents the most rapidly regenerating tissue in adult human mammals [1]. The isolation of colonic crypts and their cell types allows them to mimic native colonic tissue via the formation of colonoids. These provide an optimal model to study the regeneration processes, general metabolism, and homeostasis of different cell types within the colon.

The colonoids contain the same cell types as the natural mammalian colon [2] and resemble its complex structure, including the stem-cell bearing crypts. Colonoids were first isolated from human healthy/cancerous colonic stem cells [3]. The intestinal stem cells, which are needed to maintain regeneration and homeostasis, are located at the base of the crypts and characterized by *Lgr5* expression [4]. These cells divide and give rise to newly generated cells, which undergo cell migration via the lateral crypt, away from the crypt base to the surface epithelium, where they perish by apoptosis after a relatively short time. The differentiation involves proliferating transit-amplifying (TA) cells, which differentiate into the mature cell types of the intestinal epithelium (goblet cells, enterocytes, enteroendocrine cells, and Tuft cells) [4].

The high suitability of colonoids as an ideal model for the natural colon, as well as for colon diseases such as inflammatory bowel disease (IBD) or colorectal cancer, has been widely explored [5,6,7,8,9,10]. Additionally, colonoids might replace native tissue for therapeutic reasons in the future. For instance, during induced colitis in a mouse model, injected colonoids were able to bind to the respective extracellular matrix of the affected site [11]. This finding reveals the enormous developmental and regenerative potential of the colonoids and underlines the need for a better understanding of their regenerative pathways.

A multitude of signaling cascades and molecules play important roles within the colon and in the cultured colonoids. The well-known *Wnt* pathway is key to regulating proliferation of the different cell types [12], as well as maintaining stem cells [3,13], by high *Wnt* concentration at the bottom of the crypt. The reduction in the *Wnt* concentration consequently triggers the differentiation of the proliferative cells into differentiated cells [14,15]. A gradient of *Wnt* concentration results in a proliferation gradient [2], which makes it possible to divide the individual crypts or the whole colonoid into a crypt zone (CZ), a proliferative zone (PZ), and a differentiated zone (DZ).

While colonoids represent a good model to track the response of such an in vitro system to external factors, those factors have to be well defined. Chemical treatment or ionizing radiation allow no spatial selectivity to treat specific cells in a colonoid. Knockdown or knockout models target all cells, which express the gene of interest. Therefore, these techniques do not allow study of single cell damage in the different colonoid zones. Such single-cell damage can help to reveal the influence of tissue lesions in the colon epithelial barrier, which might trigger problems in the homeostasis and inflammation of the gut. In the colon, damaged cells are shed into the lumen to preserve tissue homeostasis. However, excessive repeated damage can lead to failure of the epithelial barrier. We propose the use of laser-based nanosurgery to reach defined ablation of single cells to study colonoid regeneration in a three-dimensional setting.

Femtosecond laser systems are suitable for nanosurgery in cellular systems [16,17]. The non-linear interactions with tissue can be used to selectively remove cellular or subcellular structures [18] to investigate the reaction of the environment. Nanosurgical ablation is based on a low-density plasma of free electrons, which is produced by multiphoton ionization and leads to the breaking of bonds between molecules [17,19]. Since the density of necessary photons occurs only in the focal plane, a precise separation from the environment can be reached, with no thermal or mechanical energy being released to the surrounding cells [17,20]. Based on the complexity and cost, femtosecond laser systems have rarely been used to study regeneration in a cellular [18,21,22] or even in an organoid context.

**Figure 1:**
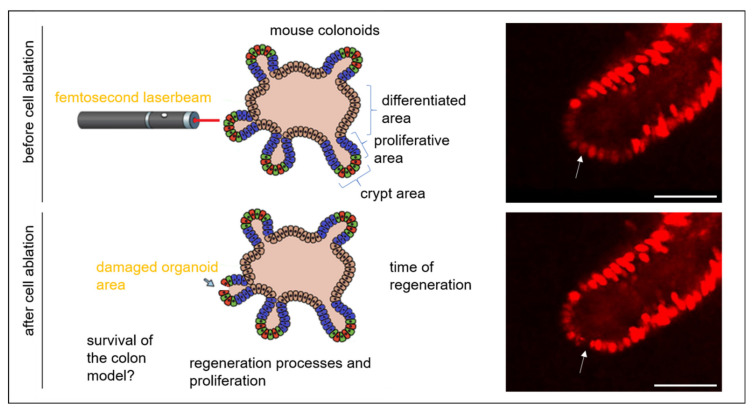
Femtosecond laser-based nanosurgery was used to remove single cells within the colonoid, which is divided into three areas, the crypt zone, the proliferative zone, and the differentiated zone. Via nuclear labeling (mCherry-H2a), the response of the colonoid to the damage of the epithelium was tracked in terms of repair, viability, and proliferation. The white arrow indicates the location of the ablated cell in a multiphoton microscopy image. The scale bar corresponds to 50 µm.

To better understand colonic regeneration, this work combines femtosecond laser-based nanosurgery to induce single cell ablation with multiphoton time-lapse imaging of colonoids. With the help of lentiviral gene transfer, cells can be marked and visualized in the colonoid (Figure 1). By precisely ablating separated cells, it was possible to investigate the behavior of the neighboring cells in relation to their localization in the colonoid over time. We observed that the three zones reacted in a different way to cell loss. Moreover, we investigated the influence on the size of the crypts, the morphology, the cell proliferation, the *Wnt* pathway via 7TGP fluorescent reporter imaging, and different proliferation associated gene expressions using RT-qPCR.

## 2. Materials and Methods

### 2.1. Isolation and Preparation of Colonoids

The protocol for the isolation of intestinal organoids was based on the work of Mahe [23]. The experiments were complied with the German Animal Protection Act (§4, TierSchG) and were approved by the local institutional advisory committee for animal care and research and by the Lower Saxony State Office for Consumer Protection and Food Safety (file number 42500/1H).

The individual mice were anesthetized with CO_2_ in an overdose at an age of 10–14 weeks. Subsequently, the neck was transected after the middle toe reflex failed to appear. The colon was exposed, separated, and transferred to ice-cold Dulbecco’s phosphate-buffered saline (DPBS) in a sterile environment. The next step was to remove the fat strands, clean the exterior, and rinse the interior of the colon with approximately 25 mL of DPBS. The colon was then cut open longitudinally with dissecting scissors, washed, and rinsed. Further reduction of the intestinal pieces into approximately 0.2 cm long sections followed, which were transferred into 15 mL crypt chelating buffer (CCB, composed of DPBS with 2 mM EDTA). A 30-min incubation in an ice bath on a rotation plate followed. After two washes with CCB, 5 mL of dissection buffer (DB, composed of DPBS containing 54.9 mM sorbitol and 43.4 mM sucrose) was added, followed by vigorous shaking. The suspension was filtered (70 µm), centrifuged (750 rcf, 5 min, 4 °C), and taken up in 720–1000 µL of Cultrex Reduced Growth Factor Basement Membrane Extract (BME), Type R1 (R&D Systems, Minneapolis, MN, USA). Then, 30 µL droplets of the suspension were plated on a 24-well plate (Costar^®^ Cell Culture Plate, Corning Incorporate™, New York, NY, USA), which had previously been preheated at 37 °C for 30 min. After 30 min of gel hardening, the droplets were covered with 500 µL organoid growth medium, composed of DMEM, high glucose, GlutaMAX™, pyruvate (Thermo Fisher Scientific, Massachusetts, MA, USA) with 50% L-WRN-supernatant (ATCC^®^ CRL3276™ in DMEM, high glucose, GlutaMAX™, pyruvate plus 10% fetal calf serum), 1x N2 (Invitrogen, Carlsbad, CA, USA), 1x B27 (Invitrogen, Carlsbad, CA, USA), 50 ng/μL recombinant mouse epidermal growth factor (Sigma-Aldrich, St.Louis, MO, USA), 10 μM Y-27632 (Tocris, Bristol, UK), and 1x Cellshield (Biochrom, Berlin, Germany) and cultured at 37 °C and 5% CO_2_ [24].

### 2.2. Colonoid Culture and Transduction

Intestinal organoids were cultured in growth medium at 37 °C and 5% CO_2_, as described above. In the first seven days after isolation and in the first five days after transduction, the concentration of the ROCK inhibitor Y-27632 (Tocris, Bristol, UK) was increased from 10 µM to 20 µM. Ten days after isolation, the colonoids were genetically modified via lentiviral transduction. The lentiviruses were produced via a third or second generation split packaging protocol in 293T cells (DSMZ, Braunschweig, Germany), as previously described [25]. One of the two transfer plasmids contained a sequence for the expression of a fusion protein from histone 2A and mCherry under the control of a CMV promotor. The other transfer plasmid was used to express a *Wnt*-sensitive promoter with seven TGP repeats that leads to fluorescence by EGFP synthesis [26]. 7TGP was a gift from Roel Nusse (Addgene plasmid #24305; http://n2t.net/addgene:24305 (accessed on 14 June 2021); RRID: Addgene_24305). For lentiviral transduction, 12 wells of 10-day-old colonoids from a 24-well plate were transduced with lentiviral particles produced from 4 × 10 cm dishes (5 × 10^7^ IU). The protocol for lentiviral transduction of intestinal organoids was described previously by van Lidth de Jeude [27].

### 2.3. Laser Setup, Imaging, and Manipulation

A Chameleon Ultra II laser system (Coherent Inc., Santa Clara, CA, USA) with a pulse length of 140 fs and a repetition rate of 80 MHz was used for multimodal imaging and manipulation of the colonoids. Using half-wave plates (Thorlabs, Newton, MA, USA) and polarizing beam splitter cubes (Thorlabs, Newton, MA, USA), the power of the laser was adjusted, depending on the application. The control of two galvanometer-scanning mirrors and a shutter was realized via a LabView (National Instruments, Austin, TX, USA) based application (LLS Rowiak, Hannover, Germany). A 40× water immersion objective with an NA of 1.2 was used to focus the laser beam into the sample. For long-term imaging of treated colonoids, an incubation chamber was added to the setup, which allowed a constant temperature of 37 °C and stable CO_2_ gas content of 5%. The colonoids labeled by the histone 2A and mCherry fusion protein were visualized at an excitation wavelength of 730 nm. The emission was detected via a photomultiplier tube (Hamamatsu Photonics, Japan) with an emission filter at 607 ± 18 nm. NADH autofluorescence was detected at 460 ± 20 nm. For nanosurgery, a wavelength of 730 nm, a pulse energy of 0.9 nJ, and a dissection speed of 10–15 µm/s was applied. Parameters for single cell ablation were confirmed by manual analysis of several 3D time series in H2A-mCherry labeled organoids (see Supplementary Videos S1 and S2).

### 2.4. Analysis of Cell Viability and Cell Proliferation

For analysis of cell viability, about 30 colonoids were placed on a glass-bottom dish (μ-Dish 35 mm, high Grid-500 Glass, Ibidi, Graefelfing, Germany), and laser ablation of single cells was performed the following day. Another 30 colonoids in a separate dish remained untreated as control. Laser ablation was performed on random locations of the organoid to obtain a general overview of the apoptosis and necrosis rate of the colonoids. A single cell was ablated according to the parameters in Section 2.3. The organoids were incubated at 5% CO_2_ and 37 °C for 24 h. Afterwards, the cells were washed and the basement membrane extract was dissolved according to the Cultrex^®^ 3D Culture Cell Harvesting Kit protocol (Trevigen, Gaitherburg, MD, USA). The cells were centrifuged for 5 min at 850 rcf. As preparation for the following steps, cells were resuspended in 4 mM EDTA in PBS. Cell viability was analyzed via flow cytometry afterwards. Cells were stained using Pacific Blue™ Annexin V staining Kit (Biolegend, San Diego, CA, USA) for analysis of dead cells according to the manufacturer’s protocol. Flow cytometric analysis was performed using a flow cytometer (Gallios™, Beckmann Coulter, Brea, CA, USA) and Kaluza Analysis 1.3 software (Beckmann Coulter, Brea, CA, USA).

The protocol for proliferation analysis is based on the studies employing EdU of Hong [28] and Oladeinde [29]. EdU is a membrane-permeable thymidine analog that can be incorporated into DNA during the S phase of the cell cycle, indicating de novo DNA synthesis. An ethynyl group is linked to the EdU. Via this nucleophilic C-C triple bond, a copper-(I)-catalyzed alkyne-azide cycloaddition (CuAAC) occurs with an azide group linked to a fluorescent molecule, forming a 1,5-disubstituted triazole conjugate. Added sodium ascorbate catalyzes the formation of Cu^+^-ions from Cu^2+^-ion sources, such as copper sulfate, which are required for coordination of the transition complex during triazole formation [28,29,30].

To study the proliferation within the colonoids, about 30 colonoids were placed on a glass-bottom dish (μ-Dish 35 mm, high Grid-500 Glass). The following day, the medium was renewed, supplemented with 10 μM EdU, and the colonoids were incubated for 2.5 h at 37 °C and 5% CO_2_. Afterwards, single cells from the crypt zone, proliferative zone, and differentiated zone were ablated from the colonoids. Imaging was performed with a power of 100 mW using two-photon microscopy. The colonoids were incubated for five more hours after the ablation process and fixed afterwards. For this purpose, old medium was removed and replaced by the fixation solution (4% Roti^®^-Histofix supplemented with 1% glutaraldehyde), which remained on the matrix drop for 20 min. The fixed colonoids were washed several times with DPBS and subsequently permeabilized by 0.5% Triton-X in DPBS treatment for 20 min. As a final step, the reaction mix (1 mM Cu_2_SO_4_, 10 mM sodium ascorbate, 8 µM 5-FAM Azide diluted in DPBS) was added to the gel drop. The fixed colonoids were incubated for one hour in the dark at 37°C and 5% CO_2_. This was followed by three washing steps overnight with DPBS on a gentle rotary shaker. The next day, colonoids were imaged on a Leica TCS SP5 confocal laser scanning microscope. Transmittance images and images of EdU staining were recorded. Next to laser treated colonoids, control colonoids which were not laser treated but otherwise treated equally were analyzed. The EdU assay was performed as described and proliferative cells were counted in defined sections. Thus, a crypt was divided into the lower section called crypt area. This opened up from the center of the bottom of the crypt to the end of the curvature within the transition to the proliferative zone. This proliferative zone starts at the end of the crypt area to 50 µm in the direction of the differentiated zone. This distance proved to be purposeful, since in most cases the end of this 50 µm showed a strong decrease in EdU-positive cells, which is thus in accordance with the present cell types. The end of the proliferative zone is followed by the differentiated zone. This procedure was performed on a high number of controls to eliminate the variation in the size of individual crypts. In ablation experiments, proliferative cells were counted 50 µm around the ablation site.

### 2.5. RT-qPCR

For RT-qPCR analysis, the colonoids were passaged on the previous day. To ensure approximately equal cell numbers, one to two wells of a 24-well plate were disrupted including the BME, Type R1 via cold DPBS, and colonoids were transferred to a glass bottom dish. Sixty individual colonoids were pulled out of the gel suspension under a transmitted light microscope using a 10 µL pipette and separated into BME of a treatment dish and a control dish. The BME was solidified at 37 °C and 5% CO_2_ for 30 min and then covered with 500 µL of organoid growth medium. The following day, a single cell was removed from each crypt of each colonoid in the treatment dish using femtosecond laser-based nanosurgery. Three biological replicates were examined, each consisting of 30 colonoids that exhibited a prominent crypt structure. The laser treated and separate control colonoids were incubated afterwards for 4 h. The cells were washed according to the Cultrex^®^ 3D Culture Cell Harvesting Kit protocol (Trevigen, Gaitherburg, MD, USA), the basement membrane extract was dissolved, and the cells were centrifuged. Cells were resuspended in DPBS, centrifuged and lysed using the Luna^®^ Cell Ready Lysis Module (New England Biolabs, Ipswich, MA, USA). The lysates were stored at −20 °C and used the next day for qPCR analysis, using the Luna^®^ Universal One-Step RT-qPCR Kit (New England Biolabs, Ipswich, MA, USA) and a TOptical Thermocycler (Biometra, Analytik Jena, Jena, Germany). Here, 1.5 µL of lysate was used in each case and a duplicate determination was performed. As a control, 30 untreated, equally passaged colonoids were washed, harvested, and lysed in parallel. Primer sequences and gene names are listed in Appendix B, Table A1. The qPCR results were processed according to the method of Tayler et al. [31].

### 2.6. Image Analysis and Statistics

All recorded image data were analyzed via Fiji [18]. For the analysis of the data, the colonoids were divided into three areas. Individual time-lapse images of the crypt, proliferative zone, and differentiated zone were manually screened to analyze cell dynamics. Proliferative, EdU positive cells were counted manually in the ablation plane. The crypt area extended from the bottom of the crypt to the point where the curvature of the crypt changes to a straight-line axis towards the colonoid. From this point, the proliferative zone was defined to extend 50 µm along the axial direction. After this distance, the differentiated zone begins, which also extends over 50 µm. In ablation studies, the proliferative, green fluorescent cells were counted to a distance of 50 µm around the ablation site, which is located in one of the three separated zones. Data were analyzed and graphically represented in OriginPro 2019 (OriginLab Corporation, Northampton, MA, USA). Either a *t*-test or a One-Way ANOVA was used to test for significant differences between groups. In all cases, *p*-values < 0.1 were considered statistically significant. For all experiments, treated colonoids were randomly selected and at least three independent dishes per experimental condition were used. Data are presented with the number of analyzed colonoids *n* and standard deviation (SD) or standard error of the mean (SEM).

## 3. Results and Discussion

### 3.1. Colonoid Viability Is Unaffected by Cell Ablation

**Figure 2:**
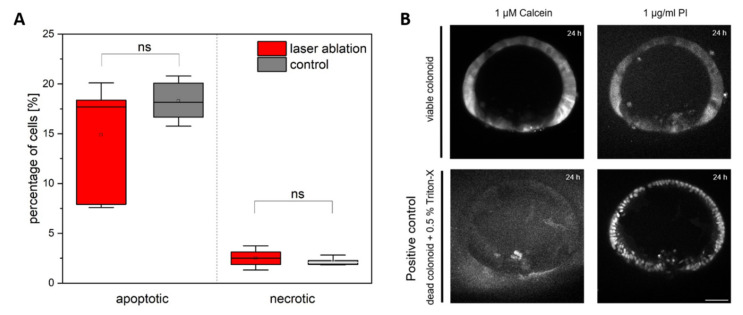
Influence of single cell ablation on cell survival in colonoids. (**A**) Apoptosis and necrosis in laser treated organoids via FACS (*n* = 6 with ~ 30 organoids per experiment, box-whisker). The rate of apoptosis is in the range of 15–20% in both laser treated and control organoids without significant differences. The rate of necrosis is in the range of 2.5% without significant differences between both groups. (**B**) Staining of a colonoid with positive Calcein signal and negative specific Propidium Iodide (PI) 24 h after laser ablation of a single cell confirms the high cell viability in laser treated organoids, compared to a control group which was permeabilized using Triton-X. Scale bar 50 µm.

Based on the enormous regeneration potential of the colonoids, we hypothesized that the cells in the organoid survive laser-based ablation of single cells. We randomly ablated a single cell from the colonoids and investigated cell viability (apoptosis and necrosis) via FACS analysis. The localization of the ablation site was also set randomly in these experiments, to obtain an overview of colonoid regeneration after laser ablation (see Figure 2A). No significant differences were found between treated and control organoids for both apoptosis (*p* = 0.18, *t*-test) and necrosis rate (*p* = 0.33, *t*-test), indicating that laser-based single cell ablation does not negatively affect the organoid’s cell viability. This was also confirmed by Calcein AM and Propidium Iodide staining 24 h after laser ablation.

Additionally, we were interested in the structural integrity of the whole colonoid. To evaluate this, the integrity of the cell layer compared to untreated samples was evaluated. NADH autofluorescence was imaged over time. NADH-related autofluorescence is in general associated with stress, apoptosis, necrosis, or general damage to the cell [32,33,34]. This is also reflected in the high autofluorescence signals that occur in the lumen of colonoids because of naturally dying cells. Intrinsic, or autofluorescence, even represents an indicator of cell viability, as increasing levels of autofluorescence correlate with non-viable cells [35,36].

**Figure 3:**
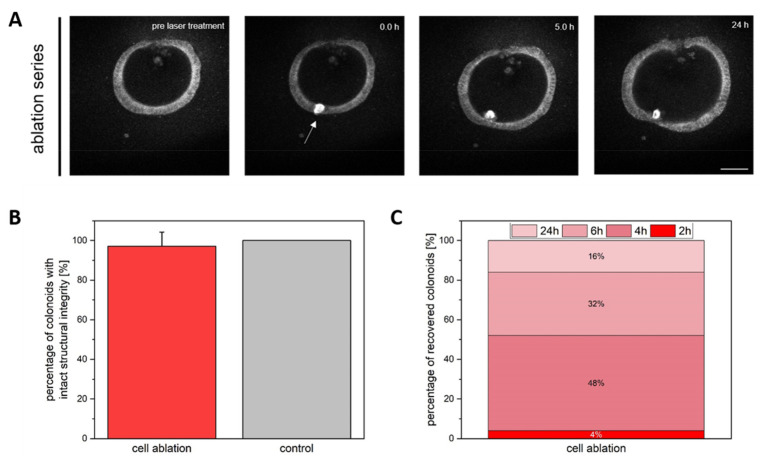
Influence of single cell ablation on the structural integrity of the colonoids. (**A**) A representative time series of cell ablation. Ablation initially leads to strong autofluorescence in the manipulated region. Subsequently, an invagination of the ablated cells occurred and the cell layer closed over time. (**B**) 97.6% of damaged colonoids survived single cell ablation (*n* = 125, SD). In the control group (*n* = 64, SD), the survival rate was 100%. (**C**) The stacked bar chart indicates the percentage of recovered colonoids in defined time intervals. Scale bar 50 µm.

**Figure A1:**
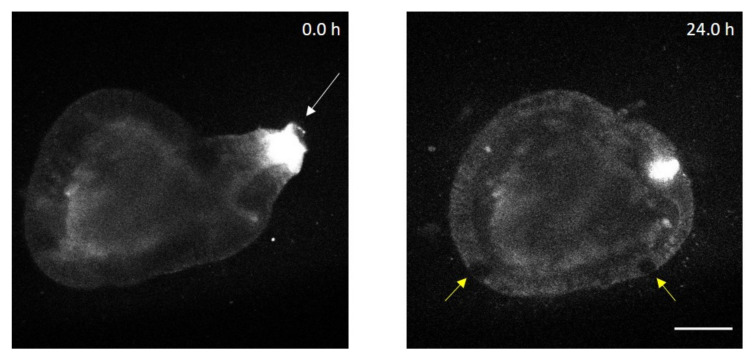
Two-photon microscopy of a colonoid that failed to repair single-cell ablation. The white arrow shows the ablation site, which has not resealed over time, even after 24 h. The cell layer of the colonoid shows gaps (yellow arrows) and no intact integrity. Scale bar 50 µm.

Removal of a single cell via laser ablation barely affected the structural integrity of the colonoids over time. At the damage location, strong autofluorescence could be observed (Figure 3A). The autofluorescence in the targeted cell is caused by the destruction of the mitochondria, which releases various molecules such as NADH and NADPH, as well as photosensitizers such as porphyrins, which have absorption bands in the UV [17,37,38]. This autofluorescence sometimes extended the ablated cells. This might be caused by photostress, which cannot be fully excluded during irradiation [39]. Overall, more than 97% of the colonoids regained their structural and cellular integrity after single cell ablation (Figure 3B). This was assessed on the basis of colonoid morphology, cell morphology (intact cell outlines), the presence of defined autofluorescence, and the integrity of the cell layer. To provide a better understanding of this classification, Appendix A, Figure A1 shows a colonoid that could not regenerate the cell layer. The cell layer of the colonoids closed over time and NADH autofluorescence was maintained. Based on a measurement of the cell layer thickness and the time for closing the colonoids, we concluded that about 84% of colonoids already recovered within 6 h (Figure 3C).

**Figure A2:**
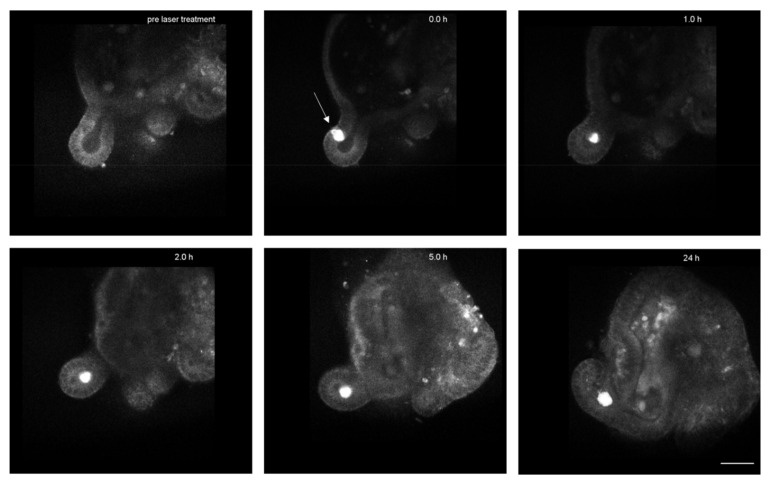
Time series of a single cell ablation in an unstabilised colonoid. The ablation site is labeled with a white arrowhead. One hour before cell ablation, the treated samples were removed from the BME, washed, and placed in a new dish to sink. In the progress of the regeneration and proliferation process, the morphology around the ablation site and throughout the colonoid changes significantly compared to stabilised colonoids. Scale bar corresponds to 50 µm.

Additionally, the structural support of the surrounding extracellular matrix could potentially influence colonoid regeneration. To test this, we detached colonoids from their extracellular matrix before laser ablation. In this case, 91% of the intestinal organoids, which received single cell ablation, survived. In the control group, the survival rate was 97%. The colonoids, which were no longer anchored in BME, showed a strongly altered morphology after 24 h (see Appendix A, Figure A2). This was the case for both controls and treated samples. Despite this altered morphology, the colonoids exhibited comparable survival rates to BME-anchored and laser-treated colonoids, which, in contrast, showed similar morphology to untreated samples.

Based on these results, it can be postulated that colonoids can repair single cell ablation at a high ratio, independent of the anchorage in the BME, which reflects the enormous regenerative potential of the natural colon. Nevertheless, anchorage is important for the basic homeostasis of the colonoid, as the extracellular matrix provides important support for various proliferation, cell division, and colonoid growth processes.

### 3.2. Single Cell Ablation Did Not Increase Proliferation in the Differentiated Zone of Colonoids

**Figure A3:**
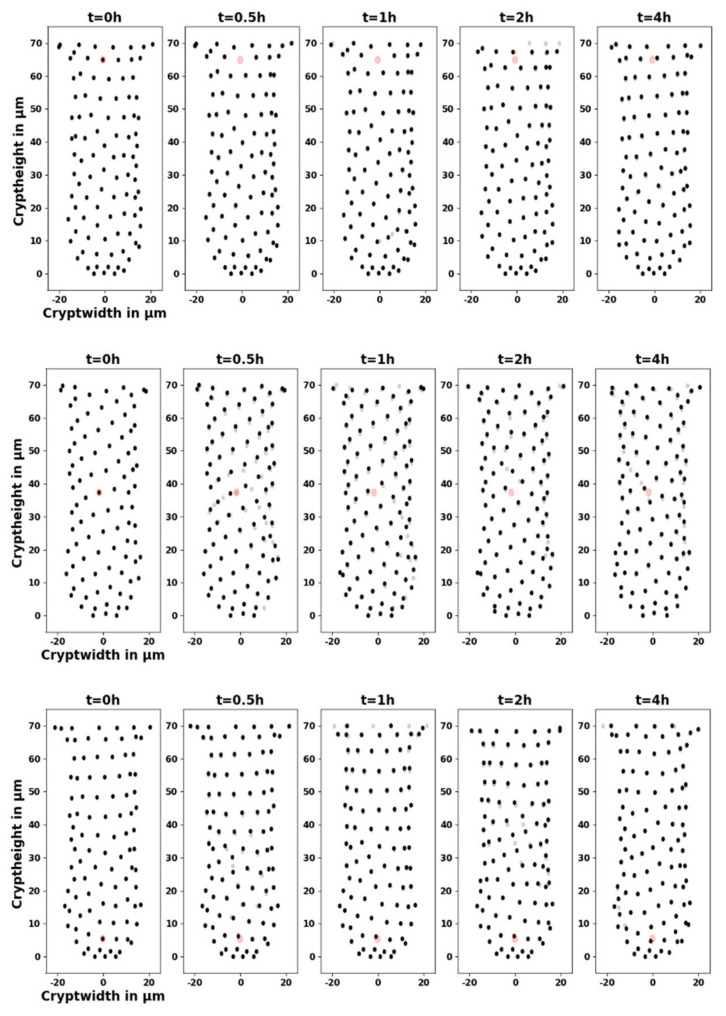
Simulation of cell removal in a colonic crypt zone. The simulation was performed using CHASTE [40,41,42], which is an agent-based simulation. We employed a previous colon crypt model in which the cell type and duration of the cell cycle are dependent on the *Wnt* concentration at birth. The *Wnt* concentration decreases linearly from the maximum value of 1.0 at the crypt base. The crypt zone ranges from 0–10 µm, the proliferative zone from 10–40 µm, and the differentiated zone from 40–70 µm. The graphs show the cell movement after ablation of a cell in the three relevant crypt areas. The individual cells are shown as black dots, the gray dots are the cell positions from the previous time point, and the red dots show the position of the cells that are ablated at the first time point. In all cases, neighboring cells enter the ablation space.

**Figure A4:**
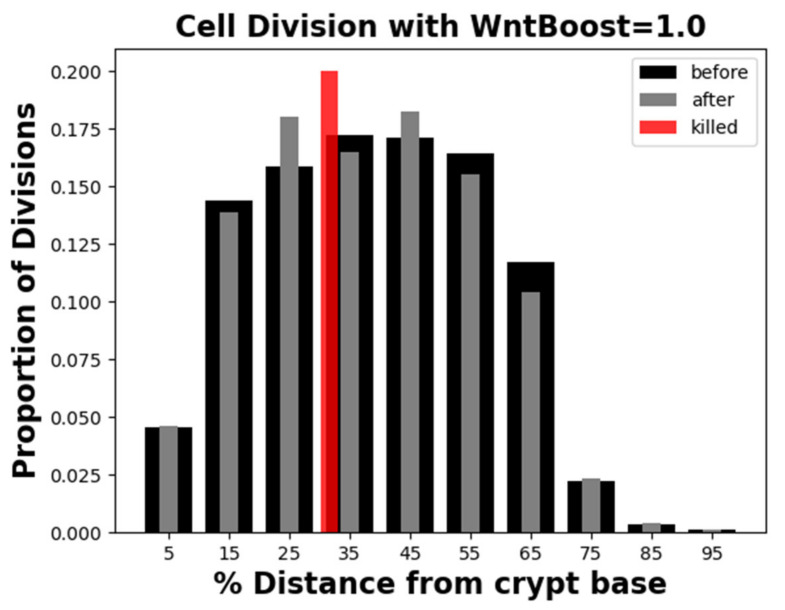
We employed the same CHASTE [40,41,42] model to probe whether a small local *Wnt* increase would result in globally visible proliferation changes. After ablation of a single cell in the crypt zone, the *Wnt* concentration of the neighboring cells was increased by 1.0. The simulation was run 10 times and averaged over the results. The percentage of cell division is plotted versus the distance from the crypt base. In black is the cell division before ablation and in gray after ablation. The averaged position of the ablated cell is shown in red.

To gain a better understanding of the regeneration processes initiated by laser ablation, the treated colonoids were classified into three groups, where laser ablation was performed in the crypt zone, the proliferative zone, or the differentiated zone. The classification was based on the cell types present and the properties associated with these cell types [4]. The definition of the differentiated zone was based on the spatial distance from any crypt bases. Furthermore, we observed fluorescently (mCherry-H2A) labelled cell nuclei and used the EdU assay to evaluate potential proliferation. We hypothesized that the damage-induced gap within the cell layer was closed either by migration of existing cells or by triggered local proliferation. This question was also studied by simulation of a colonic crypt using an already published model in CHASTE [40,41,42]. In the model, removal of a single cell led to movement of neighboring cells inside the damaged region (see Appendix C, Figure A3 and Figure A4). Our results indicated that ablation of a single cell in all zones could lead to an invagination of the cell in the damage region and moving of neighboring cells upwards to fill the gap (Supplementary Video S1). Additionally, cases without invagination were observed (Supplementary Video S2).

**Figure 4:**
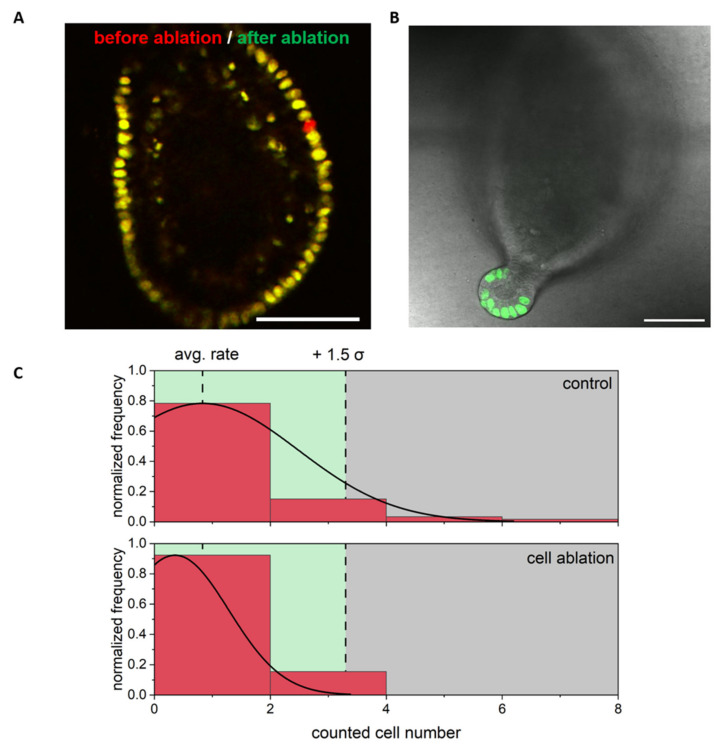
Regeneration and proliferation after ablation of a single cell in the differentiated area. (**A**) Overlay of an image before (red) and after (green) cell ablation. Only the missing cell is red colored. (**B**) Exemplary stack image (confocal microscopy stack merged of EdU and transmittance images) from the same colonoid as shown in A after fixation and EdU staining, showing no EdU positive cells outside of the crypt. (**C**) Frequency of counted EdU positive cells: In the differentiated zone proliferation was not increased and no statistical differences between control and ablation group were observed. Scale bars 50 µm and *n* ≥ 13 colonoids. The full 3D dataset to B is provided as Supplementary Video S3.

Potential cell proliferation in the differentiated region was determined within a radius of 50 µm around the ablation site, with an average of 15 visualized nuclei (Figure 4). The proliferation rate was not increased (Figure 4B,C) and no statistical difference compared to control colonoids was observed via EdU cell count (*p* = 0.3, *t*-test with *n* ≥ 13).

These results are consistent with the predominant cell types and their characteristics, as they are already fully differentiated and thus should not indicate strong, active proliferation, even after a cell ablation.

### 3.3. Single Cell Ablation Did Not Increase Proliferation in the Proliferative Zone of Colonoids

Next, we were interested if single cell ablation in the proliferative zone can trigger cell proliferation to repair the damaged region.

**Figure 5:**
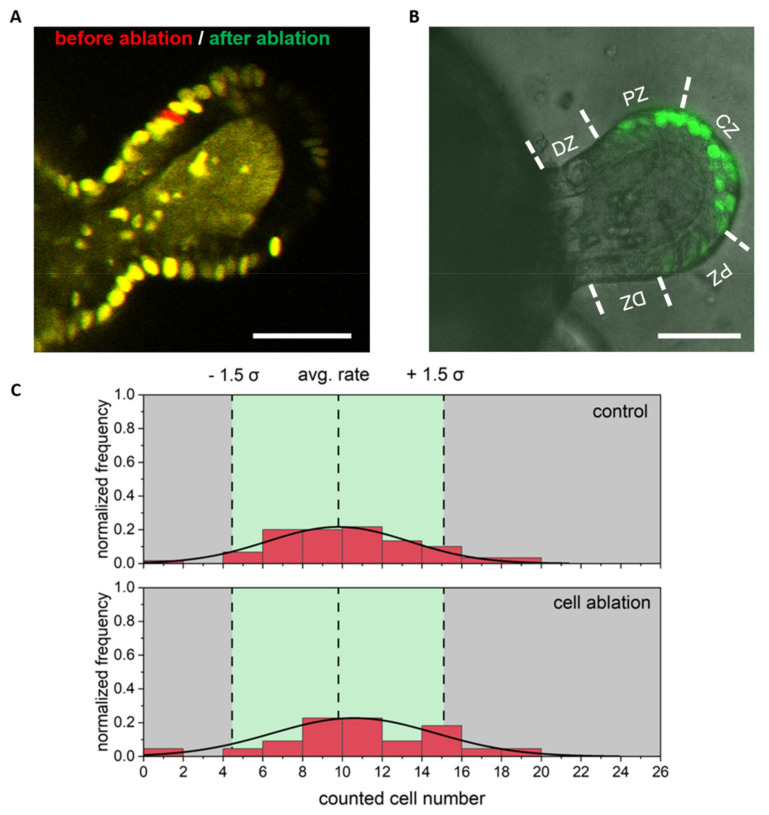
Regeneration and proliferation after ablation of a single cell in the proliferative area. (**A**) Overlay of an image before (red) and after (green) cell ablation. (**B**) Exemplary stack image from the same colonoid as shown in (**A**) after fixation and EdU staining, showing many EdU positive (green) cells in this area. The differentiated zone (DZ), proliferative zone (PZ), and crypt zone (CZ) were delineated from each other by dashed line. (**C**) Frequency of counted EdU positive cells: In the proliferative zone, proliferation (based on counted, positive EdU cells) in all samples and no statistical differences between control and ablation group were found. Scale bar 50 µm and *n* ≥ 21 colonoids. The 3D dataset to (**B**) is provided as Supplementary Video S4.

Again, migration of neighboring cells to the damaged region and an invagination of ablated cell mass were observed. The general proliferation rate in the proliferative zone was ten times higher compared to the differentiated zone. However, we observed no statistical difference between colonoids, which were laser manipulated, and untreated colonoids (*p* = 0.39, *t*-test with *n* ≥ 21, Figure 5).

### 3.4. Single Cell Ablation Can Induce Two Different Scenarios in the Crypt Zone of Colonoids

Based on the presence of stem cells and various other cell types and signaling pathways, the crypt is the most interesting area to study regeneration as a response to local damage. Choi et al. [43] analyzed the small intestinal crypt in vivo with localized laser damage. They observed rearrangement of preexisting cells, without any cell division in the crypt within two hours. Furthermore, a dilation of the crypt lumen, which forced damaged cells out of the crypt, was found [43].

**Figure 6:**
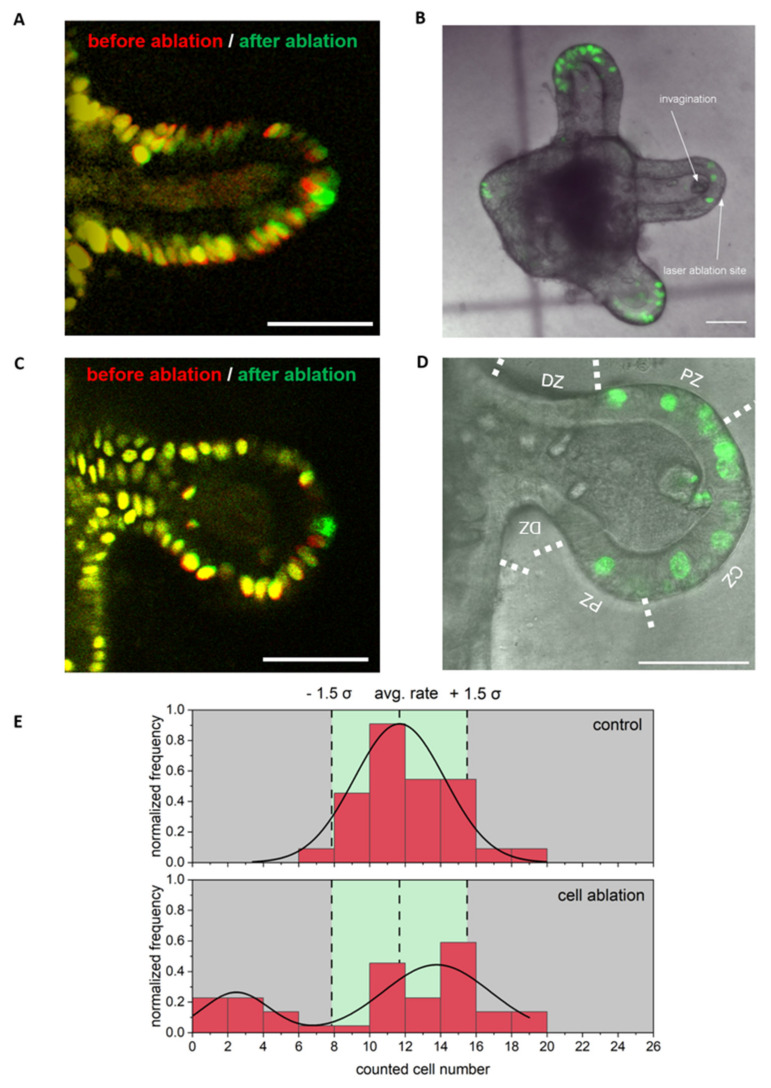
Regeneration and proliferation after ablation of a single cell in the crypt. (**A**) and (**C**) Overlay of an image before (red) and after (green) cell ablation. (**B**) Z-projection (84 µm, z-step size 2 µm) of the confocal stack of EdU and transmittance images from the same colonoid as shown in (**A**) after fixation and EdU staining, showing a decreased number of EdU positive (green) cells in the ablated area compared to two control crypts. (**D**) Z-projection (104 µm, z-step size 2 µm) of a confocal stack of EdU and transmittance images from the same colonoid, as shown in C, after fixation and EdU staining, showing many EdU positive (green) cells and the invagination of the ablated cell mass in this area. The differentiated zone (DZ), proliferative zone (PZ), and crypt zone (CZ) were delineated from each other by dashed line. (**E**) The frequency of EdU positive cells (green), indicating the proliferation rate points to two scenarios. Either normal proliferation occurs or proliferation within the crypts and the proliferative area is almost completely prevented in comparison with neighboring crypts. Scale bars 50 µm and *n* ≥ 49 colonoids. The 3D dataset to (**B**) and (**D**) is provided as Supplementary Videos S5 and S6.

In agreement with Choi et al., treated colonoids showed quick rearrangement and moving of neighboring cells in our experiments and an invagination of damaged cells. However, we observed two cases concerning proliferation: Some colonoids (~27%) showed no or a decreased proliferation after laser ablation in the crypt zone (Figure 6A,B,E), while, in the majority of colonoids, proliferation was of a similar rate to the control group (~73%, Figure 6C–E). Overall, the observed proliferation after single cell ablation in the crypt zone was significantly different compared to control colonoids (*p* = 0.07, *t*-test with *n* = 49).

**Figure 7:**
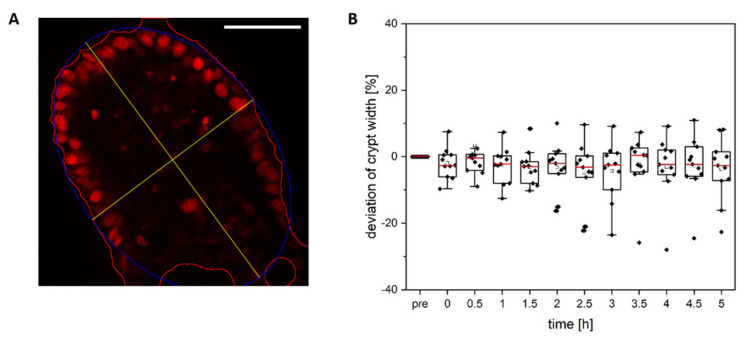
Analysis of the crypt form. (**A**) Using a self-written Fiji macro, which approximates the crypt circumference using an ellipse, the dependence of the crypt width after single cell ablation was determined over time. (**B**) The crypt form was not significantly altered over time (*n* = 11). Scale bar 50 µm.

To determine whether ablation of cells from the crypt zone is also associated with a crypt dilation, as described by Choi et al. [43], we measured the form of all crypts using a custom written ImageJ macro (Figure 7A,B). We observed no statistically significant differences in the crypt form over time after ablation (*p* 0.76, *n* = 11, One-Way ANOVA).

### 3.5. Local Wnt Pathway Changes after Single Cell Ablation in the Crypt Zone of Colonoids

Both canonical and non-canonical *Wnt* signaling pathways play important roles in intestinal regeneration in response to injury [44,45]. To further investigate the regenerative or proliferative response of crypts to damage, crypt cells were ablated from colonoids, which were transduced using a *Wnt* reporter [26].

It contains a *Tcf/Lef*-sensitive promoter with EGFP reporter to focus on the canonical signaling pathway via the β-catenin cascade [13,46].

**Figure 8:**
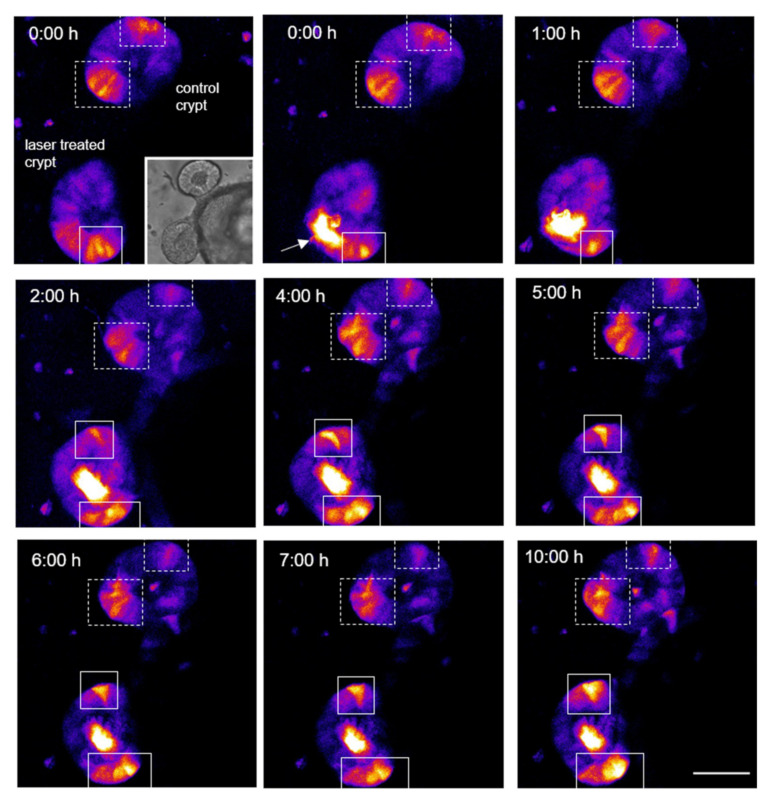
Time course of a regeneration process after ablation of a cell within the crypt area leads to an increased *Wnt* pathway signal around the ablation site. The fluorescence signal of a z-projection (confocal microscopy, 52 µm, z-step size 2 µm) of two crypts of the same colonoid (top row left, small icon) is shown. The fluorescence signal is triggered by a promotor, which controls the expression of an EGFP and is sensitive for transcription factors of the *Tcf/Lef*-family [26]. A cell was ablated in the lower crypt zone (indicated by the arrow, strong autofluorescence); the upper crypt serves as a control. Increasing fluorescence in the neighboring cells (white boxes) in the treated colonoid, as depicted in this representative time series, was observed in 7 out of 14 ablated crypts. The upper control crypt showed a fluorescence signal at a constant level (in the white, dashed boxes) over time. Scale bar 50 µm. Supplementary Video S7 shows the time course with additional comments.

Ablation of a single cell within the crypt zone resulted in an increase in EGFP fluorescence approximately 2 h after ablation (Figure 8, crypt in the left-hand corner). The fluorescence continues to increase up to 10 h after ablation, whereas the rise of the signal in the unablated control crypt is absent. Based on the continued increase it could be clearly differentiated from autofluorescence, which occurs immediately due to laser irradiation (see Section 3.1). The beginning of increase around 2 h is also in agreement with other studies using *Tcf/Lef* based reporters, which already showed reporter activation 1 h after *Wnt* activation, especially considering the short maturation time of EGFP [47,48]. The increased EGFP fluorescence occurred mainly at the edge of the crypt zone. This scenario of increasing fluorescence signal was observed in seven out of fourteen ablated crypts.

We further investigated the role of a local *Wnt* increase using computational modelling via CHASTE [40,41,42]. An already published model of the colonic crypt [40] was modified to increase the local *Wnt* concentration in neighboring cells after removal of a single cell in the crypt zone. No detectable changes in cell proliferation were observed in the model (see Appendix C, Figure A3 and Figure A4). Therefore, we were interested in whether local changes in a crypt can induce changes in the colonoid’s gene expression.

### 3.6. Single Cell Ablation in the Crypt Zone Changes Expression Levels of Different Proliferation and Wnt Associated Genes

**Figure 9:**
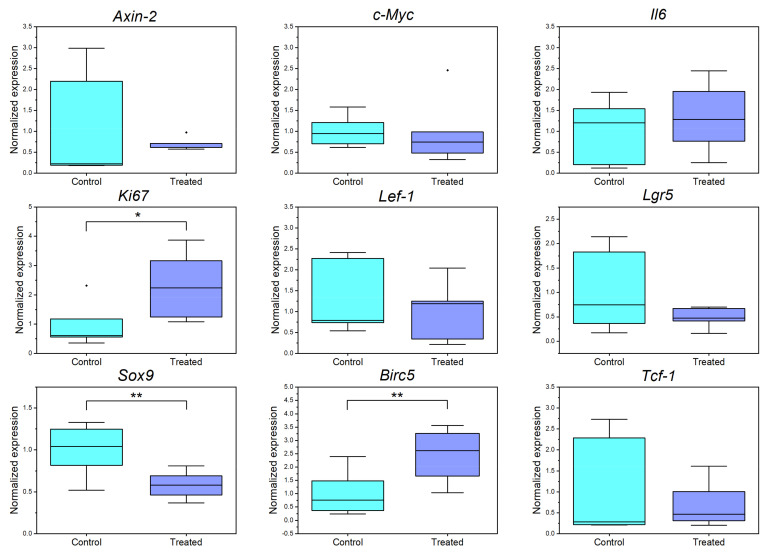
Box-plots of normalized gene expression levels of inflammatory (*Il6*), *Wnt*-associated (*Axin-2, c-Myc, Lef-1, Sox9, Tcf-1*), proliferation (*Ki67, Birc5*), or stem cell associated genes (*Lgr5*). Gene expressions were examined 5 h after laser ablation (4 h incubation at 37 °C and 5% CO_2_, 1 h colonoid harvesting and lysing). A single cell from each crypt region of 30 colonoids was ablated. Thirty untreated colonoids per dish served as controls. A duplicate assay of three biological samples and two technical replicates was performed. The respective gene expression levels were related to the expression of the housekeepers *Gapdh* and *β-actin*. * *p* ≤ 0,05, ** *p* ≤ 0,01.

To better understand the interrelated pathways, downstream targets of the *Wnt* signaling, and proliferation associated gene expressions, we performed RT-qPCR analysis (Figure 9).

In each case, a single cell was ablated from every crypt zone of 30 colonoids per dish. The treated colonoids were incubated for 4 h and then subjected to one-step qPCR analysis.

Based on our previous observations, the proliferation markers *Ki67* [49] and *Survivin* (*Birc5*) [50] were selected to investigate the effect of single-cell ablation on the expression of proliferation associated genes. Since 73% of all ablation scenarios showed comparable proliferation to control colonoids (compare Figure 7C–E), no global decrease was expected. On the contrary, a significant increase in the expression levels of *Ki67* and *Birc5* was observed in the ablated colonoids (Figure 9). We hypothesize that the increase in gene expression of these markers occurred largely in untreated, neighboring cells. *Ki67* is expressed at different levels in all non-quiescent cells [51], and is consequently not an ideal proliferation marker for our purpose, as cells might remain at a distinct cell cycle point with high *Ki67* expression after ablation. Therefore, most of our analysis was built upon EdU staining, which can only enter the nucleus during the S-Phase and serve as a binary proliferation marker [51,52]. Nevertheless, the significant increase in expression level in response to damage within the crypts indicates a role of the *Ki67* protein potentially associated with proliferation.

Next to the proliferation markers, we were looking for changes in gene expression levels in *Wnt*-pathway associated genes, for example *Sox9*, which is expressed in the colon crypt zone in the untreated case. *Sox9* partially inhibits the *Wnt* pathway via supporting phosphorylation of β-catenin in the nucleus [53,54] to avoid excessive proliferation in the stem cell area. We observed a significant downregulation in the *Sox9* expression level as a result of a localized damage in the crypt zone.

In addition to the significant changes shown, no alteration was detected in other genes involved in the *Wnt* pathway, such as *Axin-2* [55], *c-Myc* [56], or *Tcf-1/Lef-1*. This may be caused by too small expression changes, which were submerged in the overall expression of the colonoids in qPCR. In the future, spatial transcriptomics, for instance, via sequential fluorescence in situ hybridization [57], might help to shed light on localized gene expression changes.

Furthermore, the expression of *Il6*, an inflammatory marker, was analyzed. *Il6* is involved in immediate answer to injury and infection [58]. Again, no significant change could be detected in qPCR analysis, which can be attributed to low changes in the global colonoid expression pattern. The same result was found in the expression levels of the stem cell marker *Lgr5* [1].

## 4. Conclusions

The combination of single cell ablation using a femtosecond laser and colonoids possesses huge possibilities for investigating regenerative and proliferative properties of the gut. It allows for elucidation of which mechanisms contribute to proliferation-associated repair of damage in the crypt zone of colonoids. We showed that damage in the crypt region can also lead to proliferative arrest. Potential reasons might be that either different cell types have been laser ablated or biomechanical differences in the crypts exist, which needs better characterization and cell marking techniques in future studies. Additionally, we observed an increase in an EGFP-based *Wnt* report indicating *Wnt* activation. On a global expression level, *Ki67* was upregulated, which might point to increased proliferation. Additionally, *Survivin (Birc5)*, which is involved in intestinal healing, proliferation, and apoptosis inhibition, was upregulated [59,60]. In future studies, the local increase of *Wnt* signaling in response to crypt zone damage needs to be further investigated. An experimental separation of proliferative and non-proliferative crypts after laser damage followed by transcriptomic analysis would be of high interest for future analysis. This might be reached by using a live cell fluorescent cell-cycle sensor that could also allow investigation of potential continuation of proliferation in organoids with proliferation arrest. Single cell sequencing connected to the cell phenotype [61] might be a way to accomplish this goal and to align global expression data to local changes.

Our model with localized damage can also be expanded to further organoid-biology fields. For instance, other cells, such as immune cells, can be included to study their response to damage. Additionally, organoids challenged by microorganisms or from disease-specific phenotypes could be investigated to reveal potential changes in regenerative dynamics.

## Data Availability

All data can be obtained from the authors upon reasonable request. Please contact kalies@iqo.uni-hannover.de.

## Supplementary Materials

The following supporting information can be downloaded at: https://www.mdpi.com/article/10.3390/cells11071143/s1. Video S1: Regeneration and proliferation after ablation of a single cell with invagination into the lumen. The colonoid expresses mCherry labeled Histon2A, which nucleus associated fluorescence signals are shown. Time course of colonoid ablation over 16 µm (−8 to +8 µm and ablation plane) and five hours is visualized. A dual color coding (right side; red= before, green= after) is used to visualize the differences before and after ablation at all time points. Scale bar 50 µm. Video S2: Regeneration and proliferation after ablation of a single cell without invagination. The colonoid expresses mCherry labeled Histon2A, which nucleus associated fluorescence signals are shown. Time course of colonoid ablation over 16 µm (−8 to +8 µm and ablation plane) and five hours is visualized. A dual color coding (right side; red = before, green = after) is used to visualize the differences before and after ablation at all time points. Scale bar 50 µm. Video S3: Merge of confocal microscopy from EdU and transmittance images after five hours post single cell ablation. The data represent the whole 3D set, which was used for Figure 4B, which shows a representative plane. Another crypt could be shown in positive z direction, which leads to additional EdU signals. The fixed colonoid is shown over 84 µm with a step size of 2 µm. Scale bar 50 µm. Video S4: Merge of confocal microscopy from EdU and transmittance images after five hours post single cell ablation. The data represent the whole 3D set of Figure 5B, in which a single, representative plane is shown. The fixed colonoid is shown over 72 µm with a step size of 2 µm. Scale bar 50 µm. Video S5: Merge of confocal microscopy from EdU and transmittance images after five hours post single cell ablation. A single cell was ablated in the right crypt (see Figure 6B). The data represent the whole 3D set, used for the z projection of Figure 6B. The fixed colonoid is shown over 82 µm with a step size of 2 µm. Scale bar 50 µm. Video S6: Merge of confocal microscopy from EdU and transmittance images after five hours post single cell ablation. The data represent the whole 3D set of Figure 6D, in which a single, representative plane is shown. The fixed colonoid is shown over 102 µm with a step size of 2 µm. Scale bar 50 µm. Video S7: Commented time course after cell ablation in Wnt-biosensor (7TGP-EGFP) expressing cells.

## Appendix B

**Table A1:** Examined genes and used primers to study expression changes in mouse colonoids by quantitative RT-PCR.

Gene	Primer Forward 5′-3′	Primer Reverse 5′3′
***c-Myc***	TCGCTGCTGTCCTCCGAGTCC	GGTTTGCCTCTTCTCCACAGAC
***Tcf-1*** ***Lef-1***	CAAGGCAGAGAAGGAGGCTAAG ACCTACAGCGACGAGCACTT	GGCAGCGCTCTCCTTGAG GGGTAGAAGGTGGGGATTTC
***Axin2***	CTGCTGGTCAGGCAGGAG	TGCCAGTTTCTTTGGCTCTT
***Sox9***	GAGCCCGATCTGAAGAAGGA	GCTTGACGTGCGGCTTGTTC
***Birc5***	TCCCGGCATGCTCTGC	TCCGCCATTCGCTCTGG
***Ki67***	AATCCAACTCAAGTAAACGGGG	TTGGCTTGCTTCCATCCTCA
***Lgr5***	CCTACTCGAAGACTTACCCAGT	GCATTGGGGTGAATGATAGCA
***Il6***	TAGTCCTTCCTACCCCAATTTCC	TTGGTCCTTAGCCACTCCTTC
